# The Complex Role of Branched Chain Amino Acids in Diabetes and Cancer

**DOI:** 10.3390/metabo3040931

**Published:** 2013-10-14

**Authors:** Thomas M. O’Connell

**Affiliations:** LipoScience Inc., 2500 Sumner Blvd, Raleigh, NC 27616, USA; E-Mail: Thomas.oconnell@liposcience.com; Tel.: +1-919-256-1155; Fax: +1-919-256-1039

**Keywords:** cancer, diabetes, insulin resistance, branched chain amino acids, mTORC1, cachexia

## Abstract

The obesity and diabetes epidemics are continuing to spread across the globe. There is increasing evidence that diabetes leads to a significantly higher risk for certain types of cancer. Both diabetes and cancer are characterized by severe metabolic perturbations and the branched chain amino acids (BCAAs) appear to play a significant role in both of these diseases. These essential amino acids participate in a wide variety of metabolic pathways, but it is now recognized that they are also critical regulators of a number of cell signaling pathways. An elevation in branched chain amino acids has recently been shown to be significantly correlated with insulin resistance and the future development of diabetes. In cancer, the normal demands for BCAAs are complicated by the conflicting needs of the tumor and the host. The severe muscle wasting syndrome experience by many cancer patients, known as cachexia, has motivated the use of BCAA supplementation. The desired improvement in muscle mass must be balanced by the need to avoid providing materials for tumor proliferation. A better understanding of the complex functions of BCAAs could lead to their use as biomarkers of the progression of certain cancers in diabetic patients.

## 1. Introduction

Diabetes and cancer are among the most prominent global public health concerns of our time. The rise in diabetes is driven in part by the growing obesity epidemic occurring in many countries around the world. A growing body of evidence demonstrates that type 2 diabetes comes with a significantly increased risk for the development of certain types of cancers [1]. Early studies revealed associations with pancreas and liver cancer and more recent studies found increased incidence of endometrial, breast, colorectal, bladder and kidney cancers [2]. In fact, the conditions of overweight and obesity which are highly associated with type 2 diabetes are estimated to contribute to 15%–20% of all cancer deaths in the US [3]. The metabolic derangements that accompany diabetes profoundly affect energy metabolism across the whole body including pancreas, muscle, adipose tissue and liver. It is well known that amino acid levels are distinctly perturbed in obese and diabetic subjects [4,5,6]. Recent studies have shown that these perturbations are especially profound in the branched chain amino acids (BCAAs) [7,8,9,10,11,12,13,14]. In a recent study by Wang, it was demonstrated that elevated levels of branched chain amino acids are a significant risk factor for the development of insulin resistance and diabetes [8]. Many types of cancer are accompanied by significant changes in amino acid metabolism due to the demands of the tumor and its interaction with the host [15,16]. Cancer patients often experience a significant amount of involuntary weight loss and muscle wasting known as cachexia. [17] In this state, supplementation with BCAAs has been studied as a way to improve outcomes by supplying materials to stimulate protein synthesis [18]. This poses a conundrum for optimizing the levels of BCAAs since the needs of the body must be balanced with the proliferative demands of the tumor while simultaneously avoiding the potential diabetogenic effects. The goal of this review is to examine some of the complex biochemical mechanisms involving BCAA metabolism and the potential roles they play in diabetes and cancer.

Comprehensive metabolite profiling has contributed greatly to the understanding of diabetes and cancer over the last decade [14,19,20]. This approach involves the use of advanced analytical platforms such as nuclear magnetic resonance (NMR) spectroscopy and mass spectrometry (MS) to detect a wide range of metabolites whose concentrations are altered in these disease states. A global profiling approach can be taken in order to detect changes in as much of the metabolome as possible. Alternatively a targeted approach can be taken such that specific metabolites or metabolite classes are interrogated. The latter is often applied when an underlying hypothesis has been proposed which includes the involvement of specific metabolites. Both global and targeted approaches have been applied to diabetes and cancer metabolomic studies. The goal of these studies is to provide further mechanistic understanding of these disease states and to search for diagnostic and prognostic biomarkers to guide therapeutic interventions.

In addition to their role in metabolic processes, there is a growing body of literature demonstrating that the BCAAs are active in cell signaling pathways including regulatory functioning in protein and lipid synthesis, cell growth (both regulated and unregulated) and autophagy [21,22,23,24,25]. Metabolite-driven derangements in these signaling pathways have been shown to play a role in insulin resistance and tumor progression. The coupling of metabolite profiles with knowledge of cell signaling pathways has led to significant insight into how these metabolic derangements lead to progression of these disease states.

## 2. BCAAs in Bioenergetics and Protein Synthesis

**Figure 1:**
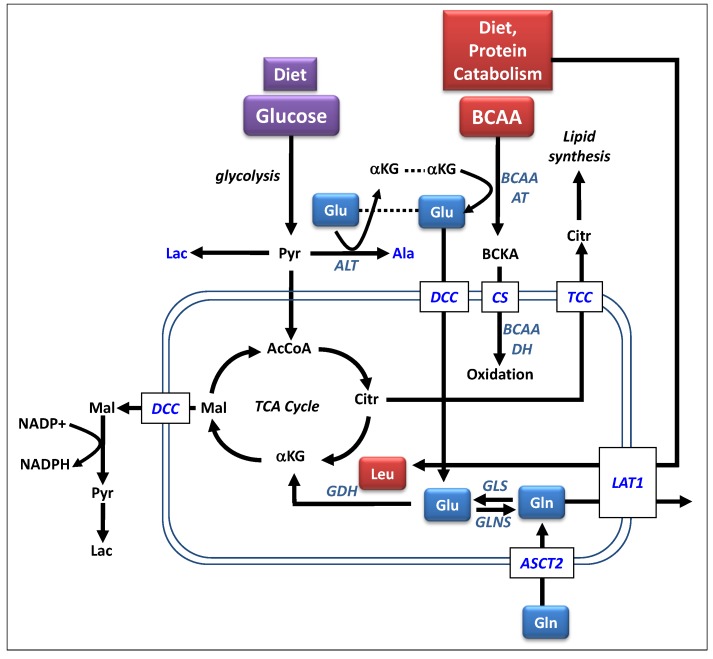
Metabolic pathways involved in branched chain amino acid metabolism. Abbreviations: αKG, α-ketoglutarate; AcCoA, acetyl-CoA; Ala, alanine; ALT, alanine aminotransferase; ASCT2, ASC-type amino acid transporters; BCAAAT, branched chain amino acid aminotransferase; BCAADH, branched chain amino acid dehydrogenase; BCKA, branched chain ketoacid; Citr, citrate; CS, carnitine shuttle; DCC, dicarboxylate carrier; Glu, glutamate; GDH, glutamate dehydrogenase; GLS, glutaminase; Gln, glutamine; GLNS, glutamine synthetase; LAT1, Large neutral amino acid transporter; Lac, lactate; Leu, leucine; Mal, malate; NADP+; nicotinamide, adenine dinucleotide phosphate; NADPH, reduced form of nicotinamide, adenine dinucleotide phosphate; Pyr, pyruvate; TCC, tricarboxylate carrier.

In order to understand how the derangement of BCAA metabolism contributes to disease, an understanding of the normal metabolic activities is necessary. The BCAAs, composed of valine, leucine and isoleucine, are three of the nine essential amino acids for humans. They account for nearly 35% of the essential amino acids in muscle proteins and approximately 40% of the preformed amino acids required by mammals [26]. The levels of BCAAs are carefully regulated by an enzymatic system that quickly responds to conditions of excess or deficiency [27]. The catabolic pathways for branched chain amino acids are shown in Figure 1. The first step in this pathway is the reversible transamination catalyzed by branched chain aminotransferase to form branched chain ketoacids (BCKA). Skeletal muscle is considered the initial site of BCAA catabolism because of the high activity of the aminotransferase in muscle. Other essential amino acids are catabolized mainly in the liver. The BCKAs formed in the muscle can be released into the blood stream where they can be taken up by other tissues and reaminated to reform the BCAAs for protein synthesis [26]. Alternatively, the BCKAs can undergo irreversible oxidative decarboxylation by the branched chain ketoaciddehydrogenase (BCKADH) complex. This forms a series of coenzyme A compounds that can be further oxidized to ultimately form acetyl and succinyl CoA for use in the TCA cycle.

The direction of the BCAA aminotransferase reaction is influenced by a number of factors including the availability of the substrates in the coupled reactions shown in Figure 1 [26,28]. The enzymes BCAA aminotransferase (BCAAAT), alanine aminotransferase (ALT) and glutamine synthetase (GLNS) are all reversible and near equilibrium and thus any changes in substrate availability (e.g., BCAAs, alanine, glutamine, glutamate and α-ketoglutarate) results in a change in enzyme activities, including the oxidation of the BCKAs. Studies have shown that enhanced supplies of BCAAs will increase the oxidative pathway along with the release of resultant alanine and glutamine [29,30,31]. Conversely, enhanced supply of glutamine was shown to reduce the activity of the oxidative pathway.

The catabolism of BCAAs provides an important source for the generation of amino acids, especially glutamine and alanine as shown in Figure 1. Glutamine circulates with the highest concentration among all of the amino acids and serves as a major bioenergetic substrate for cell growth [32]. Glutamate is formed from α-ketoglutarate in the initial transamination step of the BCAAs to form the BCKAs. It is the primary nitrogen donor for the synthesis of non-essential amino acids and thus, is critical for cellular proliferation in both regulated and unregulated states. Glutamate can support cellular energy production by entering the TCA cycle as an anapleurotic substrate. Conversion of glutamine to glutamate by glutaminase (GLS) further supports the anapleurotic supply of intermediates into TCA cycle. Glutamate can also contribute to the formation of fatty acids via the export of malic acid from the TCA cycle. Malic acid is exported from the mitochondria and goes on to form pyruvate and lactate with the generation of NADPH, a major energy source for fatty acid synthesis. Citrate from the TCA cycle is also exported to the cytosol where it is converted to acetyl CoA by reaction with oxaloacetate. This is the starting point for fatty acid synthesis. Under normal conditions, glucose is the dominant fuel for the TCA cycle and the contribution from anapleurotic substrates such as glutamate is less significant. Under different conditions, such as cancer or proteocatabolic illnesses, the contribution of glutamine, driven in part by the availability of BCAAs, is much more significant.

Alanine is used in protein synthesis and as precursor for gluconeogenesis in the liver. The generation of alanine is coupled to the catabolism of BCAAs as shown in Figure 1. The glutamate produced in the transamination reaction goes back to α-ketoglutarate in a reaction that transforms pyruvate to alanine. Under periods of starvation, alanine can participate in the glucose alanine cycle proposed by Felig, wherein muscle BCAAs generate alanine which is transported to the liver where it is used in gluconeogenesis [33].

## 3. The Role of BCAAs in Diabetes

The link between amino acids and insulin resistance has been known for decades [4,5,6] but with the advent of comprehensive metabolomic profiling a more detailed picture of how amino acids participate in the progression of diabetes is being revealed. In a recent study by Newgard, mass spectrometry based metabolomics was applied to a cohort of 73 obese and 67 lean individuals [7]. A principal components analysis of the metabolite data was applied to search for differences between the lean and obese subjects. Several components were comprised of the expected metabolic features such as long chain fatty acids, ketones and medium chain acylcarnities. Interestingly, the first principal component, accounting for the largest amount of variance in their model was composed of the branched chain amino acids along with Glx (glutamine and glutamate), methionine, the two aromatic amino acids (phenylalanine and tyrosine) and the C3 and C5 acylcarnitines. The C3 and C5 acylcarnitines can be derived from the oxidation products of BCAAs. The increased levels of the downstream metabolites in this principal component suggest that the associations are related to an alteration in the flux of the BCAAs through this catabolic pathway. This principal component had a significant linear relationship with HOMA indicating that these metabolites are significantly associated with insulin resistance as well as obesity.

Several other studies have shown that a branched chain amino acid signature is associated with insulin resistance. In a cross sectional study, a group of sedentary metabolic syndrome subjects underwent frequent oral glucose tolerance tests to measure insulin sensitivity. Targeted profiling of 75 metabolites including amino acids, acylcarnitines and free fatty acids revealed that large neutral amino acids, including branched chain amino acids, were inversely associated with insulin sensitivity [34]). A similar targeted metabolite profiling approach was applied to a group of 263 non-obese Asian-Indian and Chinese men in Singapore. The subjects were separated into upper and lower quartiles of insulin resistance based on HOMA. After controlling for BMI, the normal risk factors for IR such as free fatty acids and inflammatory cytokines could not discriminate between the upper and lower quartiles of insulin sensitivity. The strongest correlation with IR came from a panel of metabolites including the branched chain amino acids [35].

The value of branched chain amino acids appears to go beyond just the correlation with insulin resistance. In a recent study, by Wang *et al.*, 2,422 normoglycemic individuals were followed for 12 years [8]. Over this time, 201 developed diabetes. The cases were matched with controls by age, BMI and fasting glucose. A global MS based metabolomics analysis was carried out which found that a panel of five branched chain and aromatic amino acids demonstrated a significant association with the future development of diabetes. This finding was further supported by replication of this signature in an independent prospective cohort. These findings suggest that the BCAAs may presage the development of type 2 diabetes by up to a decade or more and thus, may be among the earliest detectable metabolic derangements on the route to diabetes.

## 4. Amino Acids, Glucose and the mTORC1 Pathway

**Figure 2:**
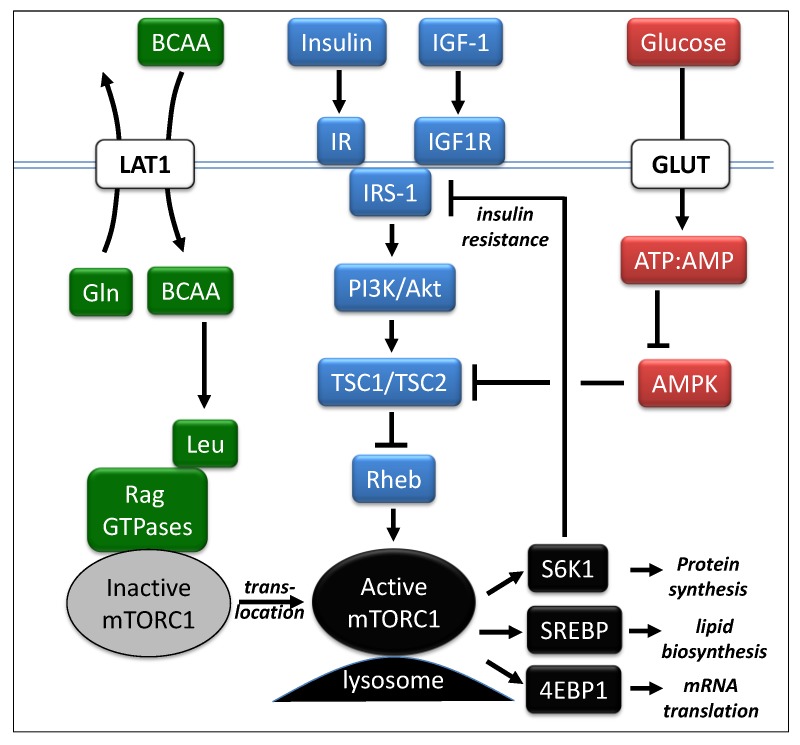
Major cell signaling pathways involving mTORC1. The activity of mTORC1 is modulated by three major pathways (Adapted from Melnik, [47]). Abbreviations: Akt, also known as Protein Kinase B; AMP, adenosine monophosphate; AMPK, adenosine monophosphate kinase; ATP, adenosine triphosphate; 4EBP1, eukaryotic translation initiation factor 4E binding protein 1; GLUT, glucose transport protein; IGF-1, insulin-like growth factor-1; IGF1R, IGF1 receptor; IR, insulin receptor; IRS-1, insulin receptor substrate 1; mTORC1, mammalian target of rapamycin complex 1; PI3K, phosphatidylinosotide 3-kinases; Rag GTPases, Rag guanine triphosphate hydrolysis enzymes; Rheb, Ras homolog enriched in brain;; S6K1, ribosomal protein S6 kinase; SREBP, sterol regulatory element-binding protein; TSC1/TSC2, complex of tuberous sclerosis proteins 1 & 2.

In obesity and diabetes, nutrient overload, especially fat consumption, has often been considered the major culprit, but increased protein intake can also contribute through elevated levels of circulating amino acids [36]. These excesses are detected by the nutrient sensitive kinase, mammalian target of rapamycin complex 1 (mTORC1), which is a master regulator of protein synthesis, lipid synthesis, gene transcription and autophagy pathways [21,22,23,24,25]. mTORC1 functions to couple nutrient sensing with cell growth and proliferation. Activation of mTORC1 is dependent upon the availability of sufficient concentrations of amino acids, especially the branched chain amino acids [37,38]. The activity of mTORC1 is modulated by three major pathways as shown in Figure 2. One pathway involves growth factors such as insulin and insulin-like growth factor 1 (IGF-1). These activate the PI3K/Akt complex leading to phosphorylation of tuberous sclerosis protein 2 (TSC2) which attenuates the inhibitory function of the TSC1/TSC2 complex [39,40]. This inhibition leads to the activation of the small GTPase, Ras homolog enriched in brain (Rheb), finally leading to activated mTORC1 [41]. Downstream of mTORC1 are at least three critical factors that contribute to cellular proliferation. Activation of the ribosomal protein S6 kinase 1 (S6K1) leads to protein synthesis [42] , activation of SREBP leads to increased lipid synthesis [43,44] and inhibition of 4EBP1 enables transcription [45]. The activation of S6K1 also yields a negative feedback loop on the insulin receptor substrate 1 (IRS-1) leading to an attenuation of insulin sensitivity [46].

Glucose levels also impact the activity of mTORC1 via the AMP kinase (AMPK) pathway. When glucose levels are high, ATP levels rise and AMP levels fall, leading to an inactivation of AMPK. Like the PI3K/Atk complex, AMPK interacts with the TSC1/TSC2 complex leading to the downstream activation of mTORC1 [40,48]. When AMPK is inactive, as would be expected with the high glucose levels present in the high glycemic Western diet, the inhibitory function of the TCS1/TSC2 complex is mitigated leading to downstream activation of mTORC1 with resultant insulin resistance.

The branched chain amino acids, especially leucine, have been associated with the stimulation of skeletal muscle protein synthesis via mTORC1 [37]. Studies in mammalian cells have demonstrated that amino acid deprivation can rapidly abolish mTORC1 activity [49]. It has been recently shown that leucine promotes the translocation of inactive mTORC1 to the lysosomal compartments which contain activated Rheb [50]. This comes about through another example of the interplay between the BCAAs and glutamine. Leucine participates in this process by directly binding to and activating glutamine dehydrogase (GDH) [51]. This leads to the production of α-ketoglutarate which is the primary effector of the recruitment of inactive mTORC1 to the lysosome. Leucine has also been shown to interact with the AMPK pathway. In a study using rat skeletal muscle, elevated concentrations of both leucine and glucose were found to decrease AMPK kinase activity, increase protein synthesis and mTORC1 activity and cause insulin resistance [45]. These effects were all mitigated by the addition of pharmacological inhibitors of AMPK. This suggests that a common mechanism exists wherein glucose and leucine modulate mTORC1 activity, protein synthesis and insulin resistance.

## 5. BCAAs in Cancer

Over the past 30 years, a growing body of epidemiological studies have indicated that obese and diabetic individuals have a far higher risk of getting some types cancer than lean, healthy people and when they do get it, their prognosis is much worse [52]. The strong link between obesity and insulin resistance suggests that the metabolic pathways involved in insulin resistance and diabetes are at play in this dramatically increased cancer risk. A number of studies have demonstrated that significant alterations are found in the utilization of BCAAs in cancer and in the associated cachectic wasting disorder [18,53]. Understanding the metabolic perturbations that arise in cancer demands an appreciation for the energetic and proliferative needs of both the host and the tumor.

The most fundamental metabolic change that occurs in cancer cells is an increased reliance on aerobic glycolysis known as the Warburg effect [54]. In normal cells, pyruvate generated by glycolysis is carried into the mitochondria where it undergoes oxidation. In cancer cells, even in the presence of sufficient oxygen, pyruvate stops short of the TCA cycle and is transformed to lactate. Glycolysis alone is not an efficient means of generating energy. The lactate formed is reconverted into glucose via the Cori cycle. This inefficient process is increased by up to 50% in cancer patients and accounts for up to 60% of the lactate production [55,56].

In order to engage in replicative division, a cell must duplicate its genome, proteome and lipidome and assemble the components into daughter cells. With the rapid, dysregulated cell growth found in cancer cells, extra sources of energy and nutrients like glucose and essential amino acids are required. It is well know that cancer cells have a greatly increased demand for glutamine [50,57]. Several studies have shown that the rates of glutamine consumption in cancer cells are substantially higher than any other amino acid with overall consumption far exceeding the energetic demands of the cell [58]. Nearly 50 years ago, Eagle observed in a study of various cancer cell lines that glutamine consumption rates of many of the cell lines exceeded those of the other AAs by more than 10 fold [59]. The up- regulation of glutaminolysis in cancer cells can provide a significant fraction of the NADPH needed for cellular proliferation. This is generated through the export of malate [60] as shown in Figure 1. The export of citrate further along the TCA cycle provides the lipids necessary for lipid synthesis and the construction of new cell membranes.

Glutamine also plays a role in the upregulation of mTORC1 to facilitate the proliferative processes in tumor cell growth. The export of glutamine to the extracellular space via the L-type amino acid transporters (e.g., LAT1) is coupled with the import of essential amino acids, especially leucine [38]. The leucine then functions as described earlier to activate mTORC1 by initiating cellular translocation to the lysosome. The increased demands of cellular proliferation in cancer are met by the downstream transcription factors show in Figure 2 [43,44].

## 6. BCAAs in Cachexia

One of the most devastating sequela of many types of cancers is cachexia. This is a complex wasting disorder that accompanies many chronic diseases and has a profound effect on whole body metabolism. It is a major contributing cause of morbidity and mortality in up to 50% of patients with advanced cancer [17]. Cachexia is characterized by massive loss of both adipose and skeletal muscle tissue which reduces the quality of life and the efficacy of many chemotherapeutic interventions. The mechanisms involved in the development and progression of cachexia are very complex, but alterations in amino acid metabolism appear to play a major role [61]. Cancer patients are often anorexic due to the disease or the treatment so reduced dietary intake of amino acids likely plays a role. The progression of cachexia appears to involves a net increase in protein catabolism along with activation of proteolysis and a significant reduction in protein synthesis.

Under normal conditions, BCAA oxidation in skeletal muscle provides 6%–7% of the energy needs, but under highly catabolic circumstances such as cancer cachexia, the contribution can be as high as 20% [62]. Myofibrillar proteins are composed of approximately 18% BCAAs and therefore, breakdown of skeletal muscle can yield significant increases in BCAAs [53]. A number of studies have noted widespread decreases in circulating amino acids in cachectic patients, with the most profound decreases being for the BCAAs [63,64]. It might be expected that the increased proteolysis would lead to an increase in the circulating BCAAs, but this increase leads to an increase in the oxidative pathway. The tumor itself also uses the catabolized BCAAs along with the other amino acids for protein synthesis. It should be noted that the balance of catabolism, oxidation and protein synthesis may be quite different for different tumors at different stages and thus, the observed BCAA levels may be difficult to interpret without a greater metabolic and phenotypic profile.

An elevated inflammatory state has been noted in proteocatabolic states such as cancer cachexia. Chronic inflammation affects amino acid availability due to the cytokine stimulated hepatic synthesis of acute phase proteins. These cytokines have also been demonstrated to induce BCAA oxidation by activating BCKADH [65].

## 7. Therapeutic Use of BCAA Supplementation

In treating a patient with cancer cachexia, therapeutic strategies could involve a protein deficient diet to starve the tumor or a protein rich diet in order to mitigate the devastating cachectic wasting. Given the unique demands of skeletal muscle on BCAAs, a number of studies have been conducted to investigate the utility of specific BCAA supplementation to inhibit protein catabolism and stimulate protein synthesis and potentially muscle repair [66,67,68]. Selective supplementation of BCAAs could positively affect the protein synthesis requirements of the host while providing insufficient amino acid resources for tumor proliferation.

The effects of BCAA supplementation on the induction of insulin resistance should be considered in any therapeutic strategies. Following on the observation of the BCAA signature of insulin resistance, Newgard *et al.*, conducted animal studies in which the BCAA supplementation was used to trigger insulin resistance [7]. Only in the context of a high-fat diet was the insulin resistance observed. This suggests that the overall nutritional status of an individual greatly influences the diabetogenic potential of BCAA supplementation.

Advanced liver diseases, including hepatocellular carcinoma (HCC), have received a great deal of attention regarding the therapeutic utility of branched chain amino acid supplementation. Kobayashi *et al.* [69] conducted a study of cirrhotic patients caused by hepatitis C and followed the progression to HCC over several years. In this study, there was a control group with standard nutritional supplementation and another group which received a BCAA supplement. The authors reported a lower incidence of HCC in the BCAA group. In a large scale clinical study in Japan (Long Term Survival Study; LOTUS), BCAA supplementation was evaluated for its effects on the onset of complications in cirrhosis patients including death, liver failure and HCC [70]. Compared with a control diet, the group receiving BCAA supplementation experienced fewer complications (hazard ratio: 0.67; 95% CI: 0.49–0.93). After stratifying the subjects for HCC risk, specifically patients with a body mass index greater than 25 kg/m^2^, the BCAA supplementation had an inhibitory effect on the progression to HCC. Tsuchiya reported that long term BCAA supplementation in patients who received radical therapy for HCC reduced the rate of relapses and improved the cumulative rate of survival [71].

Recent studies have revealed that BCAA supplemental therapy in patients with liver cirrhosis actually improves their insulin resistance and hyperinsulinemia [72], which can account for the reduced risk of HCC. BCAA supplementation is thought to prevent insulin resistance by promoting insulin-independent glucose uptake by skeletal muscle and improving glucose tolerance [73]. A study comparing enteral nutrition *vs.* BCAA supplementation in liver failure demonstrated improvements in the maintenance of serum albumin levels, a marker of protein synthesis [74]. An interesting finding in this study was that the enteral nutrition group experienced an increase in glycated hemoglobin and other markers of abnormal glucose tolerance while no such changes were observed in the BCAA group. This study shows that the dietary increase in BCAAs under these conditions does not lead to the same alterations in glucose homeostasis as long term elevations.

Several recent studies using cell culture methods have shed some light on specific pathways in which branched chain amino acids affect liver metabolism and tumorigenesis. In a study of hepatic stellate cells, it was found that leucine stimulated the secretion of hepatocyte growth factor (HGF) [75]. HGF is considered to be a pleotropic factor that is produced by cells in various organs and influences cell growth, function and motility [76]. Leucine-stimulated HGF could then provide a means for liver regeneration in the context of liver diseases, including HCC. A recent study using HepG2 cells demonstrated that supplementation with all three branched chain amino acids led to the degradation of VEGF mRNA [77]. This would indicate that administration of BCAAs to cirrhotic patients could potentially decrease the progression to HCC through suppression of VEGF expression. In a recent study using the H4IIE hepatic tumor cell lines, it was found that BCAAs suppressed insulin-induced cell proliferation [78]. The effect was not attributed to a reduction in cellular proliferation, but rather an increase in apoptosis.

## 8. Conclusions

Diabetes and cancer come with widespread metabolic perturbations that affect the entire body and the branched chain amino acids appear to be among the most distinctly perturbed metabolites. Given the critical role they play in a wide array of physiological processes, it is clear that there could be a great deal of clinical value in monitoring their levels. The challenge of using the BCAAs as biomarkers comes with the multitude of competing energetic and proliferative demands present in both healthy and disease states [79]. Furthermore, the stage of the disease and in the case of cancer, the location of the tumor, will also affect how the levels of BCAAs are affected. The ultimate utility for BCAAs in diagnosing, predicting or monitoring disease will depend upon the presence of additional information that will place these levels in the context of the entire phenotypic condition. This information may include routine chemistry measurements, such as glucose and insulin levels, nutritional and dietary factors, along with weight status (e.g., recent weight loss from cachexia). There is continued opportunity for comprehensive metabolite profiling to shed light on the competing processes at play in these potentially inter-related disease states.

A systems level approach will provide valuable insights into the interrelated pathogenesis of diabetes and cancer. Further genetics and proteomics studies could reveal novel signaling pathways that are affected by the endogenous and supplemental levels of BCAAs. Continued study of multiple tissues including muscle, adipose, liver and tumor using animal models and cell culture will lead to a more holistic understanding of BCAA metabolism. With a more complete mechanistic understanding, the BCAAs may be a significant component of diagnostic testing panels to monitor the development of diabetes and the associated risk of cancer. 
